# Seeking Novel Personalized and Sex-Specific Strategies for the Prevention and Treatment of Heart Failure Based on the Assessment of β1-Adrenergic Receptor Desensitization: The Contribution to the HEAL ITALIA Project

**DOI:** 10.3390/cimb48020132

**Published:** 2026-01-25

**Authors:** Rosa Vona, Camilla Cittadini, Gianfranco Mattia, Rossella Puglisi, Barbara Ascione, Lucrezia Gambardella, Sonia Maccari, Giuseppe Marano, Paola Matarrese

**Affiliations:** Centre for Gender-Specific Medicine, Istituto Superiore di Sanità, 00161 Rome, Italy; camilla.cittadini@iss.it (C.C.); gianfranco.mattia@iss.it (G.M.); rossella.puglisi@iss.it (R.P.); barbara.ascione@iss.it (B.A.); lucrezia.gambardella@iss.it (L.G.); sonia.maccari@iss.it (S.M.); giuseppe.marano@iss.it (G.M.); paola.matarrese@iss.it (P.M.)

**Keywords:** heart failure, estrogens, β adrenergic receptors, miRNA, sex differences

## Abstract

**Background:** This study is part of the HEAL ITALIA partnership, funded by the National Recovery and Resilience Plan (PNRR) and the European Union. Heart failure (HF) is a serious health problem, with a reduced density of the β1-adrenergic receptor (β1-AR) in the myocardium as a hallmark. It is unclear whether this downregulation causes dysfunction or represents an epiphenomenon. Recent evidence implicates oxidative stress and mitochondrial signaling, particularly through the 18 kDa translocator protein (TSPO), in the regulation of the β1-AR, with possible modulation by estrogen. **Objectives:** To determine (1) the role of β1-AR desensitization in the onset and development of HF; (2) whether monocytes can represent a suitable ex vivo model for sex-oriented mechanistic studies in the cardiac field; (3) whether monocytes isolated from peripheral blood of patients can represent a diagnostic and/or therapy response biomarker by monitoring β1-AR density; (4) whether and how the mitochondrial receptor TSPO is involved in the β1-AR dysregulation observed in HF; and (5) whether the mechanisms linked to the onset of HF are regulated in a sex-specific manner through the effect of estrogen and/or the X chromosome on the expression of specific microRNAs. **Methods:** Using an integrated in vitro-ex vivo-in vivo methodological approach, we will evaluate the density of β1/β2-AR receptors, the downstream signaling (GRK2/β-arrestin), mitochondrial and redox parameters, and miRNA profiles in human monocytes and cardiomyocytes, and in mouse hearts after HF following pressure overload. **Conclusions:** The goal is to better understand the mechanisms underlying β1-AR desensitization, verify monocytes as peripheral markers of disease progression and response to therapy, and provide potentially useful information for the development of gender-specific therapies for heart failure.

## 1. Introduction

### 1.1. Background

Heart failure (HF) is a large pathophysiological syndrome that results from a primary defect that affects the capability of the heart to fill or eject blood appropriately. It can be either acute and come on suddenly, or a progressive, long-term condition. Consequently, people with HF may experience serious health complications that can reduce their quality of life [1]. The number of heart failure cases worldwide is estimated to be 26 million. When the estimated number of undiagnosed cases is added in, the figure rises to 37.7 million, with increasing numbers reported every year [2]. Despite substantial advances in pharmacological and surgical therapies, the prognosis of HF remains poor, with five-year survival rates still comparable to those of some aggressive cancers. Both men and women are susceptible to HF at any age, though it is most prevalent among the elderly (over 60 years old). There are significant differences in this condition between men and women due to disparities in the pathophysiology attributed to hormonal factors and different rates of comorbidities [1,3,4]. HF arises from the complex interplay of genetic predisposition, environmental stressors, metabolic imbalances, and neuro-hormonal dysregulation, resulting in maladaptive remodeling of myocardial structure and function. The risk of developing HF can be increased by several factors, including advanced age, obesity, hypertension, diabetes mellitus, tobacco smoking, alcohol misuse, drug misuse, and exposure to certain types of chemotherapy or radiation therapy, or failure to take preventive medications [2].

Patients with HF differ in terms of ejection fraction values, the antecedent disease that led to HF, serum catecholamine levels, and the effects of pharmacological treatment with beta-blockers. Within this intricate landscape, one molecular hallmark consistently emerges across etiologies: the reduction in β-adrenergic responsiveness due to the downregulation and desensitization of β1-adrenergic receptors (β1-ARs) on cardiomyocytes.

The β-adrenergic system, which is composed mainly of β1- and β2-AR subtypes (with β1-ARs accounting for 75–80% and β2-ARs accounting for 20–25% of the β-ARs found in the heart), is the principal regulator of cardiac output under physiological and stressful conditions [5]. β1-ARs predominate in human myocardium and mediate positive inotropic and chronotropic responses through coupling with Gs proteins, activation of adenylate cyclase, and the consequent rise in cyclic AMP (cAMP) and protein kinase A (PKA) activity. Persistent activation of this signaling cascade, as occurs in chronic sympathetic overdrive, promotes receptor phosphorylation by G-protein–coupled receptor kinases (GRKs), β-arrestin recruitment, receptor internalization, and ultimately degradation or recycling. This process, initially protective against catecholamine overstimulation, contributes in the long term to the contractile failure characteristic of advanced HF [6,7].

As the β1-AR is the most powerful regulator of myocardial contraction and relaxation, considerable interest exists in the mechanisms that regulate the β1-AR response to catecholamines. However, whether β1-AR downregulation represents a primary pathogenic mechanism or merely an epiphenomenon of cardiac injury remains unresolved. Evidence from both animal models and human samples is conflicting: some studies suggest that loss of β1-AR density precedes overt cardiac dysfunction [8,9,10] while others indicate that receptor downregulation simply reflects compensatory remodeling secondary to oxidative stress and metabolic exhaustion [11,12]. Dissecting this causal relationship is crucial because β1-AR density and signaling efficiency directly influence the clinical response to β-blockers, which are a cornerstone therapy for HF with reduced ejection fraction (HFrEF) [13]. Interindividual variability in β-ARs expression or regulation could thus underlie differential therapeutic efficacy and adverse outcomes, highlighting the need for personalized prevention and treatment strategies.

Emerging findings also implicate non-catecholaminergic mechanisms in β1-AR regulation. Oxidative and metabolic stressors—such as elevated free fatty acids, hyperglycemia, and chronic inflammation—can alter receptor turnover, trafficking, and signaling independently of GRK/β-arrestin pathways [14,15]. These conditions are common in metabolic syndrome, diabetes, and obesity, which are major risk factors for HF.

Mitochondrial dysfunction appears to play a central role in this alternative regulatory axis. Mitochondria are both generators and targets of reactive oxygen species (ROS), and oxidative modifications of membrane proteins and lipids can trigger receptor internalization or degradation [16]. Furthermore, mitochondrial stress influences the cellular redox environment, calcium handling, and energy production, all of which are known to modulate β-ARs signaling cascades [17,18]. Previous research has demonstrated that the expression of β1-AR in cardiomyocytes decreases in response to increased exposure to reactive oxygen species (ROS), identifying a novel mechanism for β1-AR downregulation [19,20]. Within the mitochondrial outer membrane, the 18 kDa Translocator Protein (TSPO)—formerly known as the Peripheral Benzodiazepine Receptor (PBR)—has emerged as a key regulator of mitochondrial bioenergetics, redox balance, and apoptosis. TSPO participates in cholesterol transport, steroidogenesis, and the formation of the mitochondrial permeability transition pore. Altered TSPO expression and function have been reported in various cardiovascular pathologies, including ischemia–reperfusion injury and cardiac hypertrophy, but its involvement in β-ARs regulation remains poorly defined [21]. Our preliminary data suggest that TSPO ligands can influence cellular ROS production and mitochondrial membrane potential, potentially affecting β1-AR stability at the plasma membrane. We therefore hypothesize that TSPO acts as a mechanistic link between metabolic and oxidative stress and β1-AR downregulation, rather than representing a mere bystander of cardiac injury. However, this regulatory axis has not yet been systematically investigated under conditions relevant to HF. In this context, mitochondrial dysfunction—and its regulation by TSPO—may function as an upstream integrative hub through which neurohormonal, metabolic, and oxidative stress signals converge to modulate β1-AR stability and signaling.

Metabolic changes contribute to maladaptive remodeling, which further contributes to β1-AR dysregulation. The normal adult heart uses fatty acids, glucose, amino acids, lactate, and ketone bodies to produce ATP, whereas the failing myocardium develops a defect in the use of both fatty acids and glucose, which is associated with mitochondrial dysfunction and reduced oxidative metabolism. The accumulation of toxic lipids induces transcriptional changes, oxidative stress, PKC activation, and the inhibition of insulin signaling. It remains to be ascertained whether lipid overload and oxidative stress lead to β1-AR downregulation in cultured cells, in murine models of diabetes-related HF, and diabetic patients with HF [22,23]. This leads to alterations in insulin signaling, endoplasmic reticulum stress, apoptosis, and mitochondrial dysfunction. Saturated long-chain fatty acids, particularly palmitic acid (16:0), are lipotoxic, inducing PKC activation and apoptosis through oxidative stress [24]. It remains to be ascertained whether and how lipid overload modulates β1-AR cell surface density.

Taken together, these observations suggest that the downregulation of β1-AR may represent a convergent molecular node rather than merely an epiphenomenon of cardiac injury. Neurohormonal activation, oxidative stress, metabolic dysregulation, and mitochondrial dysfunction all converge at the level of receptor trafficking, stability, and signaling efficiency. Understanding how these pathways interact is crucial for clarifying the causal role of β1-AR remodeling in HF and identifying novel biomarkers and therapeutic targets.

Adding a further layer of complexity, sex differences profoundly shape cardiovascular physiology and pathology. Women and men differ not only in HF incidence and presentation but also in their molecular response to adrenergic stimuli. Epidemiological data reveal that women are more likely to develop heart failure with preserved ejection fraction (HFpEF), while men predominate in HFrEF [25,26]. These differences are partly attributable to the modulatory effects of estrogens, which act through estrogen receptors ER-α, ER-β, and the membrane-bound G-protein–coupled receptor GPR30 [27]. Estrogen signaling exerts protective actions on mitochondrial function, oxidative stress, and inflammation, and may counteract β-AR desensitization by regulating receptor gene transcription, post-translational modifications, and membrane localization. Nevertheless, the specific contribution of estrogen pathways to β1-AR downregulation in human immune or cardiac cells remains uncertain.

A key role in the onset of oxidative stress in HF is played by microRNAs (miRNAs), which target genes responsible for improved fatty acid substrate utilization for energy production and are eventually responsible for cardiomyocyte apoptosis. In addition, the harmful effects of X-linked miRNAs on cardiovascular disease have been observed, suggesting that sex-biased miRNA networks could play a key role in HFpEF in women [28]. On these bases, we will search for miRNAs implicated in the complex pathophysiology of cardiomyopathy by performing miRNAomic analysis on monocytes and cardiomyocytes treated as follows. Since the gender difference observed in HFpEF could also be determined by epigenetic factors, including microRNAs, we will pay particular attention to those miRNAs present in the areas of the X chromosome that escape inactivation, therefore more expressed in females [29], and to those whose expression is linked to the presence of estrogen [28].

This gender gap is particularly relevant for precision medicine, which requires a mechanistic understanding of sex-specific responses to guide tailored interventions. Integrating estrogenic modulation into the study of β-ARs and TSPO signaling may reveal new preventive and therapeutic targets that differ between sexes.

A major limitation in cardiovascular translational research is the restricted accessibility of myocardial tissue in living patients, which hampers longitudinal assessment of β1-AR regulation during disease progression and therapy. Circulating peripheral blood mononuclear cells, particularly monocytes, offer a valuable solution to this challenge. Monocytes express both β1- and β2-ARs and share key signaling components with cardiomyocytes, including GRK2 and β-arrestin, responding to catecholaminergic, inflammatory, and metabolic stimuli through partially overlapping pathways [30]. Changes in β1-AR density on circulating monocytes have been shown to mirror those observed in cardiomyocytes, suggesting their potential use as surrogate biomarkers for monitoring myocardial adrenergic remodeling. In this study, monocytes are therefore employed not as substitutes for cardiomyocytes but as complementary translational tools enabling standardized, repeatable, and clinically applicable assessment of β1-AR regulation and its modulation by TSPO-dependent mitochondrial signaling.

Taken together, these considerations support a unifying, hierarchical model in which TSPO signaling and metabolic stress act as upstream modulators of β1-AR downregulation, representing a central convergence point for neurohormonal activation, mitochondrial dysfunction, and sex-specific regulatory mechanisms. Within this integrated experimental framework, circulating monocytes provide translational readouts, while sex-dependent pathways modulate vulnerability and therapeutic responses, ultimately aiming to elucidate causal mechanisms underlying HF development and to identify novel, potentially sex-specific, biomarkers and therapeutic targets for precision prevention and treatment.

### 1.2. Project Framing

As part of the HEAL ITALIA program—Health Extended Alliance for Innovative Therapies, Advanced Lab-Research, and Integrated Approaches of Precision Medicine—Spoke 7, “Prevention Strategies,” focuses on the development of novel personalized approaches for the prevention of cardiovascular disease. Within this Spoke, Work Package 2—new personalized strategies for the prevention of cardiovascular disease—and specifically, Task 2.2—evaluation of risk factors for chronic heart failure—aims to integrate molecular, cellular, and physiological data to refine risk prediction and identify innovative molecular targets.

In line with this mission, this study addresses a critical unmet need in cardiovascular research: the lack of minimally invasive biomarkers capable of early detection of adrenergic remodeling and driving personalized therapeutic strategies, as well as sex-specific experimental models suitable for mechanistic studies.

Based on the above conceptual framework, the project focuses on the hypothesis that β1-adrenergic receptor (β1-AR) desensitization represents a convergent molecular event integrating neurohormonal activation, mitochondrial dysfunction, metabolic stress, and sex-specific regulatory mechanisms. Within this model, TSPO-dependent mitochondrial signaling is proposed as an upstream regulator of redox balance and β1-AR stability, while circulating monocytes are exploited as a marker reflecting myocardial adrenergic remodeling.

The project leverages multidisciplinary expertise in cardiology, cellular and molecular biology, and pharmacology, combining in vitro and ex vivo experiments with studies conducted in mice engineered for β1-AR, β2-AR, or both, according to harmonized protocols.

An additional challenge in cardiovascular translational research is the limited accessibility of myocardial tissue in living patients. This limitation complicates the direct measurement of β1-AR density and signaling changes during disease progression or therapy. To overcome this limitation, the project employs peripheral blood mononuclear cells, with a particular focus on monocytes, as a minimally invasive translational platform. Monocytes share essential components of β-adrenergic signaling with cardiomyocytes and can be challenged ex vivo with catecholaminergic, metabolic, and oxidative stressors, allowing standardized quantification of β1-AR regulation and its modulation by TSPO-targeting ligands [30]. Previous studies, in fact, have shown correlations between β-AR density on circulating lymphocytes and cardiac function, but methodological inconsistencies have hindered their validation as reliable biomarkers [30]. Standardizing monocyte isolation and receptor quantification procedures could therefore enable their use as a minimally invasive biomarker of adrenergic remodeling and therapeutic response.

Mechanistic validation is achieved through parallel studies in human cardiomyocyte models, including the AC16 cell line and primary cardiomyocytes derived from male and female human pluripotent stem cells (hPSCs), as well as in vivo investigations using male and female mouse models genetically engineered for β1-AR, β2-AR, or combined β1/β2-AR deletion. These complementary models enable the analysis of causal relationships between β1-AR signaling, mitochondrial dysfunction, and heart failure development under controlled metabolic and oxidative stress conditions.

Importantly, sex as a biological variable is integrated throughout the experimental design. The project explicitly investigates sex-dependent differences in β1-AR regulation, TSPO signaling, mitochondrial function, and microRNA expression, with particular attention to estrogen-dependent pathways and X-linked miRNAs. This approach is in line with the growing awareness that sex-specific mechanisms contribute to the different prevalence of different clinical phenotypes of HF observed in men and women, i.e., HF with preserved and reduced ejection fraction, respectively.

The project leverages multidisciplinary expertise in cardiology, cellular and molecular biology, and pharmacology, combining in vitro and ex vivo experiments with studies conducted in mice engineered for β1-AR, β2-AR, or both, according to harmonized protocols.

Given these premises, the project has the following objectives:

1. Validate peripheral blood monocytes as minimally invasive biomarkers for assessing β1-AR surface density and adrenergic remodeling through methodological standardization.

2. Understand whether and how TSPO modulates β1-AR density and signaling through redox- and metabolism-dependent mechanisms and assess the therapeutic potential of TSPO ligands.

3. Establish if β1-AR desensitization plays a causal role in the development and progression of HF using complementary in vitro and in vivo models.

4. Identify sex-specific regulatory mechanisms that contribute to the onset and progression of HF, i.e., those mediated by estrogen-dependent and/or X-encoded microRNAs.

Objectives 1–3 represent the core mechanistic and translational aims of the study, whereas objective 4, related to microRNA profiling and detailed sex-specific regulatory networks, is exploratory and intended to generate hypotheses for future investigations.

## 2. Materials and Methods

### 2.1. Design

This study will integrate several approaches, using (i) ex vivo model of monocytes freshly isolated from peripheral blood of healthy donors (male and female); (ii) in vitro models using the human AC16 cell line and primary cardiomyocytes derived from male and female human pluripotent stem cells (hPSCs); and (iii) in vivo model using male and female CD-1 mice knocked out for β1-AR, β2-AR, or both.

### 2.2. Human Subjects

A gap in cardiovascular translational research is the limited accessibility of myocardial tissue in living patients for studying biomarkers of β1-AR regulation. Circulating monocytes express both β1-AR and β2-AR and can be challenged ex vivo with metabolic and oxidative stressors, offering a minimally invasive window into adrenergic remodeling. On these bases, healthy donors aged more than 18 years were recruited at the Blood Transfusion Centre of Sapienza University of Rome. The study was reviewed and approved by the Ethical Committee at ISS (protocol no. AOOI-SS-4/01/2022 0000197 of 8 January 2020). The study will be conducted in accordance with the Declaration of Helsinki. PBMCs from healthy adult donors will be obtained on the basis of the Resolution of the General Director of the Umberto I Polyclinic of Rome, no. 0000722, of 7 August 2024. The project and the patients’ informed consent were approved by the Review Board of the Italian National Institute of Health, Rome, Italy. β1-AR and β2-AR surface density evaluation will be performed in at least 3 different experiments performed simultaneously on cells isolated from 2 male and 2 female healthy donors (a total of 12 subjects: 6 males and 6 females). Considering the results from our preliminary experiments on human monocytes with adrenergic drugs, for a *t*-test with Bonferroni correction for multiple comparison, a 2-fold difference between means (13% control group vs. 26% treated group) and an SD of 3, along with six biochemical assays per group, will guarantee a power of 80% using G*Power 3.1.

### 2.3. Isolation of Human Monocytes

Peripheral blood mononuclear cells (PBMCs) will be isolated by Ficoll-Hypaque gradient, purified by negative selection, and phenotyped by flow cytometry. Freshly isolated monocytes will be exposed for 24–72 h to (a) β-agonists (isoproterenol and epinephrine), (b) fatty acids (palmitate 0.4 mM ± oleate), (c) high glucose (75–300 mM), or (d) oxidative stress (H_2_O_2_ 30–90 μM).

The same donor-derived monocytes will be treated with TSPO ligands—Ro 5-4864 (agonist), PPIX (endogenous ligand), PK11195, and diazepam (antagonists)—to quantify effects on β1-AR expression, mitochondrial potential, ATP production, and glycolytic flux (Seahorse XF Analyzer, Agilent, Sanata Clara, CA, USA). These data will clarify whether TSPO activation promotes or prevents β1-AR desensitization under metabolic stress.

At the end of pharmacological treatments, monocytes will be fixed with 0.01% paraformaldehyde for 10 min and stained with FITC-conjugated anti-β1-AR or anti-β2-AR (Alomone), previously validated by our group [30]. For analyses of redox and mitochondrial parameters, apoptosis, and autophagy, living cells will be incubated with proper fluorescence dyes or specific antibodies [29,31]. Samples will be analyzed using a FACSCalibur flow cytometer (BD Biosciences Inc., San Diego, CA, USA).

### 2.4. Cell Culture

The AC16 human cardiomyocyte cell line and human pluripotent stem cell (hPSCs) have been used as an in vitro model for the study of mechanistic dissection of β-Ars-, TSPO- and estrogen-dependent signaling.

The AC16 cell line will be cultured in DMEM-F12 (Gibco, Milan, Italy), supplemented with 10% fetal bovine serum (FBS), 100 U/mL penicillin, 100 μg/mL streptomycin, and 2 mM L-Glutamine. Cells will be maintained in an incubator at 37 °C, 5% CO_2_, and 90% humidity.

The hPSC cell line will be cultured using mTeSR™ supplemented with 5% Supplement. The cells should be seeded into tissue-culture-treated ware pre-coated with Corning^®^ Matrigel^®^ hESC-Qualified Matrix (Corning, Glendale, AZ, USA). The cells should be maintained in an incubator at 37 °C, with 5% CO_2_ and 90% humidity. Once the cells reach 95% confluency, the cardiomyocyte differentiation protocol will be initiated. Three different media will be used at the appropriate times for cell differentiation, following the manufacturer’s instructions (StemCell Technologies Inc.,Vancuver, BC, Canada). After 15 days, c will be ready for testing with our treatment or for maintenance in culture with STEMdiff™ Cardiomyocyte Maintenance Medium (StemCell Technologies a). The differentiation of hPSC cells into cardiomyocytes will be monitored by semiquantitative evaluation of cardiotropin expression following labeling with a specific antibody.

The same treatment that will be used for monocytes will also be applied to both AC16 cells and hPSC-derived ventricular cardiomyocytes. Under these different experimental conditions, we will evaluate the following: the total expression and membrane density of β1-AR and β2-AR, the expression of molecules involved in the signal transduction of β-ARs (e.g., β-arrestin and GRK2), the redox and metabolic parameters (e.g., GSH, production of cytoplasmic and mitochondrial ROS, NOS, mitochondrial membrane potential, etc.), and cell death and cytoprotection mechanisms (e.g., apoptotic and autophagic pathways). In addition, immunofluorescence staining of mitochondrial and autophagic markers will be performed.

### 2.5. miRNA Analyses

Total RNAs, including small RNAs, will be purified from isolated cells or frozen tissue by using a commercial kit. qRealTime PCR will be performed by using the TaqMan Technology and snRNA U6, and an additional reference will be selected among those miRNAs uniformly and consistently expressed in the analyzed samples, which will be used for normalization. MiRNA microarray will be performed using commercial arrays. In situ hybridization will be performed by using the miRCURY LNA microRNA ISH Optimization kit.

MiRNAomic analysis will be validated by qRealTime PCR on total RNA from both monocytes and heart tissue. MiRNA-specific target gene identity will be confirmed by luciferase reporter assay in an in vitro experiment, and the expression of the relative protein target will also be analyzed in differently treated monocytes and cardiomyocytes. Cardiac tissues taken from CD-1 mice subjected to treatments with different TSPO ligands and/or β-blockers will also be analyzed in terms of miRNA expression. Therefore, we will perform a miRNAomic study on the ventricular tissue of surgical-induced HF murine model, pointing particular attention to sex variability. We will focus mainly on the miRNAs that are X-linked or estrogen-dependent, and also those found to be regulated in human cardiac specimens.

### 2.6. Animal Studies

Animal experiments will be performed according to Legislative Decree 26/14, and approved by the Service for Biotechnology and Animal Welfare of the Istituto Superiore di Sanità and by the Italian Ministry of Health (907/2025-PR, Risp. a prot. D9997.217, of 11 December 2025 and 398/2025-PR of 3 June 2025). All efforts will be made to minimize animal suffering.

Male and female C57BL/6 mice of 10–14 weeks of age will be used in this study and will be purchased from Charles River Laboratories (Calco, Como, Italy). Male and female 10–14 week-old β1-β2-AR double KO mice and β1-AR or β2-AR single KO mice, coming from the colony present in the stables of the Istituto Superiore di Sanità (Authorization D9997.202, Decree N. 398/2025-PR), will be acquired as previously described [32]. To determine the size of the animal groups (n = 6 per group) to achieve statistical significance, we performed a G-power calculation based on previous studies using antiadrenergic drugs in a murine model of cardiac dysfunction and pathological remodeling [33].

To induce cardiac hypertrophy from pressure overload in mice, we will perform experimental coarctation of the aortic arch (CoA) via transverse aortic constriction (TAC) as previously reported [33]. Pressure overload induces left ventricular hypertrophy, subsequently leading to maladaptation and heart failure. Treatments with TSPO ligands and/or different β-blockers will be performed according to the literature data.

### 2.7. Statistical Methods

To quantitatively evaluate the cell parameters, the collected data will be analyzed using a two-way ANOVA test for repeated samples, corrected for multiple comparisons using the Bonferroni procedure. All data will be verified in at least three different experiments, each performed simultaneously on cells isolated from two male and two female healthy donors (12 subjects in total: 6 males and 6 females). Taking into account the results of our preliminary experiments on human monocytes with adrenergic drugs, a *t*-test with Bonferroni correction for multiple comparisons required a twofold difference in means (13% in the control group vs. 26% in the treated group) and an SD of 3. Six biochemical assays per group guaranteed an 80% power using G*Power 3.1.

For the animal studies, the differences between the treatment groups in terms of cardiac function and remodeling will be evaluated using a two-way ANOVA, followed by a Bonferroni or Dunnett post hoc comparison test. Post hoc tests will only be conducted if the F statistic in the ANOVA achieves the necessary level of statistical significance and if there is no significant variance in homogeneity, as assessed by Bartlett’s test. The size of the animal groups (n = 6 per group) was selected based on previous studies using antiadrenergic drugs in a murine model of cardiac dysfunction and pathological remodeling [33]. For the clinical study, differences between groups will be evaluated using analysis of variance corrected for multiple comparisons using the Bonferroni procedure, or a Student’s *t*-test. In a second phase, multivariate and Cox regression analyses will be performed.

## 3. Expected Results

The main goal of drug treatment is to administer the correct therapeutic dose to each patient. Establishing the minimum effective dose of a β-blocker for the treatment of HF is quite complicated, and there are currently no international guidelines, only general guidelines to follow (pointbypoint reference). In fact, this dose is determined empirically during therapy based on the therapeutic and adverse effects observed in the patient. Currently, there are no clinically relevant surrogate endpoints for monitoring and adjusting the dosage of β-blocker drugs. Identifying non-invasive markers of therapeutic response (e.g., levels of β1-AR in monocytes or specific miRNAs) could help clinicians define the optimal dosage for each patient with HF. This would have important medical and socioeconomic implications, representing a practical breakthrough in the clinical management of the disease and offering clear benefits to the National Health Service.

The results of this study will enable us to standardize the method of isolating monocytes from peripheral blood samples and quantifying β1-β2-AR on the plasma membrane of monocytes and cardiac tissue. They will also allow us to determine the time taken for heart failure to develop in selected mouse models, and to compare the timing and manner of cardiac alteration evolution in male and female animals. Furthermore, the project will allow us to elucidate the role played by catecholamines in the downregulation of human endogenous β1-adrenergic receptors (β1-AR) associated with HF. Finally, the study will test the hypothesis that TSPO is involved in regulating β1-AR expression in both in vitro and in vivo models. Both β1-AR and TSPO ligands will be evaluated for their impact on β1-AR density in human primary monocytes, in cardiomyocyte cell lines, as well as in primary cardiomyocytes and hearts from mice. Furthermore, we will assess the ability of TSPO ligands to interfere with cardiac function in murine models of HF caused by transverse aortic constriction (TAC)-induced pressure overload.

The insights gained from this study could lead to the identification of new therapeutic and/or preventive targets, eventually sex-specific, for the treatment of HF.

## 4. Conclusions

This HEAL ITALIA Spoke-7 study protocol addresses fundamental gaps in our understanding of the role of the β-ARs and TSPO in the onset and progression of HF. Clarifying these aspects could be important for identifying innovative, specific targets. Combining in vitro, ex vivo, and in vivo approaches, the study will provide insights into the biological significance of β1-AR desensitization and mitochondrial signaling in HF and will explore the potential role of circulating monocytes both as diagnostic and/or therapy response non-invasive biomarkers and as a sex-specific experimental model suitable for mechanistic studies.

Although this project does not foresee immediate implications for clinical practice, and appears exploratory and speculative in some respects, it could provide important information of translational value in the medium and long term. The most original aspect of this project proposal, however, lies in addressing the issue of sex-specific differences at the cellular and molecular level by proposing the use of ex vivo and in vitro model systems to explore the possible biological causes underlying the gender differences observed in HF in clinical practice.

## Data Availability

The original contributions presented in this study are included in the article. Further inquiries can be directed to the corresponding author.

## Abbreviations

The following abbreviations are used in this manuscript:

PNRR	National Recovery and Resilience Plan
HEAL ITALIA	Health Extended Alliance for Innovative Therapies, Advanced Lab-Research and Integrated Approaches of Precision Medicine
HF	Heart failure
β-ARs	Adrenergic receptors beta
GRK	G-protein–coupled receptor kinases
HFrEF	Ejection fraction
HFpEF	preserved ejection fraction
TSPO	18 kDa translocator protein
ER	Estrogen receptor
GPR30	G-protein–coupled receptor
miRNAs	MicroRNAs
